# Hidden Markov models for monitoring circadian rhythmicity in telemetric activity data

**DOI:** 10.1098/rsif.2017.0885

**Published:** 2018-02-07

**Authors:** Qi Huang, Dwayne Cohen, Sandra Komarzynski, Xiao-Mei Li, Pasquale Innominato, Francis Lévi, Bärbel Finkenstädt

**Affiliations:** 1 Department of Statistics, University of Warwick, Coventry, CV4 7AL, UK; 2 Medical School, University of Warwick, Coventry, CV4 7AL, UK; 3 INSERM U935, Hospital Paul Brousse and University Paris-Saclay, Villejuif, 94800, France; 4 Department of Oncology, North Wales Cancer Treatment Centre, Bodelwyddan, LL18 5UJ, UK

**Keywords:** circadian rhythm in rest–activity, activity counts, accelerometer data, hidden Markov models

## Abstract

Wearable computing devices allow collection of densely sampled real-time information on movement enabling researchers and medical experts to obtain objective and non-obtrusive records of actual activity of a subject in the real world over many days. Our interest here is motivated by the use of activity data for evaluating and monitoring the circadian rhythmicity of subjects for research in chronobiology and chronotherapeutic healthcare. In order to translate the information from such high-volume data arising we propose the use of a Markov modelling approach which (i) naturally captures the notable square wave form observed in activity data along with heterogeneous ultradian variances over the circadian cycle of human activity, (ii) thresholds activity into different states in a probabilistic way while respecting time dependence and (iii) gives rise to circadian rhythm parameter estimates, based on probabilities of transitions between rest and activity, that are interpretable and of interest to circadian research.

## 1. Introduction

Questions of interest regarding the research of sleep–wake cycles in humans and mammals are commonly studied by measuring activity through gross motor movement where accelerometers have become a feasible and affordable way to obtain objective non-obtrusive recordings of rest–activity rhythms of free living individuals over many days [1–3]. Accelerometers measure the acceleration of the part of the body to which they are attached, often as part of a small communicative wearable device. The signal is preprocessed by the device to obtain physical activity (PA) time-series data accumulated over a specified time interval, called *epoch*. Time-series PA data from such monitoring devices are subject to circadian rhythms and are of interest to the circadian research community.

Our current understanding of circadian rhythms as a network of molecular clocks and their relevance for human health has quickly progressed [4,5] now demanding their integration into medical and care decision processes [6–8]. The circadian timing system contains a network of molecular oscillators generated by a set of specific clock genes in almost each cell of the body [9–11]. This network is coordinated by a hypothalamic pacemaker, the supra-chiasmatic nuclei (SCN), the principal circadian clock in the brain of mammals which is entrained by visual afferents and input from other brain and peripheral oscillators. Regarding the molecular genetics of circadian rhythms it is now well established that molecular clocks within the cell consist of transcriptional–translational feedback loops involving about 15 clock genes, which generate approximately 24-h oscillations in many cellular functions at cell population or whole-organism levels [12]. The master clock in the brain synchronizes the cellular clocks in all the peripheral tissues including eye, brain, heart, lung, gastrointestinal tract, liver, kidney, bone marrow and immune system. The overall system, referred to as the circadian timing system (CTS), controls several critical molecular pathways which regulate cell cycle. Mounting evidence supports a link between circadian misalignment or disruption and increased risk for an array of chronic diseases including cardiovascular disease, cancer [6], metabolic syndrome (obesity, hypertension, arteriosclerosis, diabetes) and psychiatric disorders (depression, bipolar disorder, schizophrenia, attention deficit) [13]. Circadian rhythm alterations have also been consistently associated with poor quality of life, more severe and frequent symptoms and poor overall survival in large cohorts of cancer patients [6]. Here, we are interested in modelling PA time series with a view to evaluating and monitoring the circadian rhythmicity of subjects for research in chronobiology and chronotherapeutic healthcare, in particular cancer patients receiving a chronomodulated chemotherapy while they are in their own home and in their usual environment [14]. However, the development of relevant metrics and quantifiers of circadian rhythm that can be passively obtained from mobile sensing could potentially improve the efficacy of chronotherapeutic methods applied to a wide variety of clinical conditions including bipolar disorder, where the interpersonal and social rhythm therapy (IPSRT) is designed to help patients maintain a stable circadian rhythm and sleep–wake cycle to prevent relapse [15], Parkinson's disease, stroke, epilepsy, anxiety disorders and other conditions where treatment therapies involve monitoring patients' rhythms of daily life [16].

**Figure 1.**
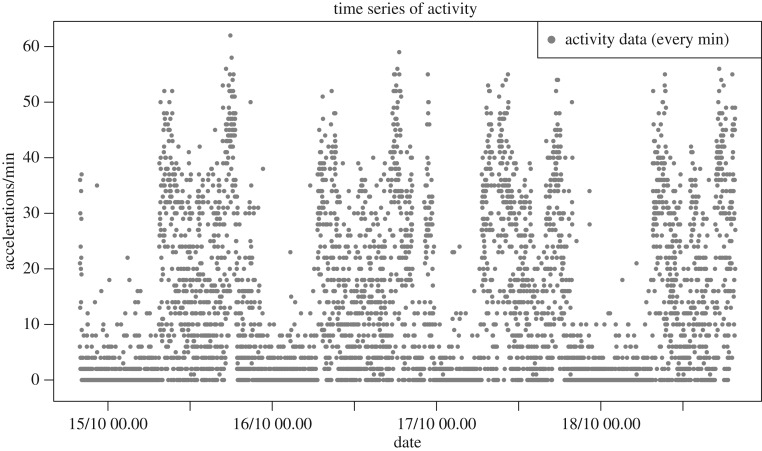
Example of raw accelerometer data: activity counts recorded per minute over 4 days with Move3 (Movisens GmbH, Germany) sensor with inbuilt accelerator ADXL345 (Analog Devices, MA, USA) fixed to the chest of a healthy individual (subject 16).

There has been a proliferation towards a multitude of wearable computing devices at increasing commercial availability and popularity. Today, activity tracking is omnipresent, usually in wrist worn devices, such as smart watches, bracelets or smart phones. The fact that they can provide frequent and non-obtrusive recording of PA in a real-world environment offers tremendous opportunities for health and makes them ideal instruments in a large variety of applications including mobile health monitoring (mhealth) [16], e.g. of chronically ill or elderly patients [17], sensing behavioural symptoms of mental health [18], self-monitoring for promoting PA levels [19,20] or studies of sentinel behaviour [21]. Wearable devices usually contain an accelerometer and, with increased computing power, more functions may be included such as heart rate sensors, ambient light sensors, temperature sensors, altimeters, etc., potentially providing very rich and complex data scenarios. Accelerometers themselves have experienced development, in particular as in 2009 the leading brand ActiGraph developed the detection of acceleration from uniaxial to triaxial. PA can now be collected at short epoch lengths, such as every minute or every 15 s, over many days. The sensor used in our study (Move3, Movisens GmbH, Germany) is fixed to the chest and contains a triaxial accelerator model (ADXL345, Analog Devices, MA, USA). The device produces activity counts defined as the number of times an accelerometer waveform computed by the device, according to specifications of the frequency and filters that are specific to the manufacturer, crosses zero over the epoch length of 1 min. Figure 1 gives an example of PA counts recorded every minute for a healthy individual over 4 days. Translating information from such high volume and complex data into interpretable and useful statistics is a challenging task, in particular if the aim is to perform long term, i.e. over many days and weeks, monitoring of an individual.

Apart from visually inspecting time plots, the data are generally analysed by deriving statistics, termed *non-parametric variables* [22,23], to quantify characteristics of interest to clinicians, sleep researchers and chronobiologists. These are generally focussed around the *intradaily variability*, which measures the disruption of the rhythm, and *interdaily variability* which quantifies the entrainment to the 24-h light/dark cycle. An R-package to compute these alongside other statistics, such as relative amplitude of activity, average activity values of the 10 h with maximal activity and the 5 h with least activity, is provided by [24]. Evidence exists in the literature [25,26] for hourly PA count data that the *intradaily variability* is a particularly important variable that is correlated with decreased sleep quality and cognitive functions as patients with Alzheimer's disease were found to have higher *intradaily variability* values [27]. In a clinical context, a series of studies [28–31] found that the *dichotomy index* I < O, which reports the percentage of epochs during the rest span when activity is lower than the median activity during wakefulness, was the most relevant statistic in predicting survival rates in cancer patients. An I < O value of 100% corresponds to a non-disrupted rest pattern and the lower the I < O value the more severe the disruption of the rest–activity rhythm. Lévi *et al.* [32] found that median survival was nearly twice as high in patients with metastatic colorectal cancer whose baseline I < O exceeded 97.5% when compared with those with a lower I < O before chemotherapy delivery [33].

While there exist numerous non-parametric variables to quantify the (mis)timing of sleep–wake rhythms, and new ones continue to be proposed [34], it is a challenging task to quantify their variability, which is important, in particular if such variables are used in assisting with the decision-making process of a health expert about an individual's therapy. An additional complication arises because most nonparametric variables discussed above rely on being able to mark the beginning and end of prolonged rest periods which, in many cases, cannot be determined unequivocally.

Fourier based methods such as the Lomb–Scargle periodogram [35], the fast Fourier transform-nonlinear least squares (FFT-NLLS) algorithm [36], harmonic regression [37] and the spectrum resampling method [38] can be used to extract further parameters, namely acrophase, amplitude and period, that are typically of interest to studies of circadian rhythmicity. Spectral estimation using the methods proposed in [38] confirms that the activity data for healthy individuals usually exhibit a strong 24-h periodicity (see electronic supplementary material, figure S1) as can be expected due the entrained endogenous circadian rhythmicity endorsed by the timing of the work and social environment. Although spectral analysis is well able to extract the circadian period, smooth functional forms, such as harmonic functions or splines, are not ideal for modelling the abrupt appearance of the transitions between the active and inactive states and will not detect short bouts of transitions caused, e.g. by daytime naps or active behaviour during nighttime. The data also show time changing variances in that PA values during the day show a markedly larger variability than over the prolonged rest period. The marginal histogram (see electronic supplementary material, figure S2 (b) for an example) displays two modes, namely a sharp peak around zero, which corresponds to the non-active period, and a wider second mode corresponding to the active period. Here we shall propose the use of hidden Markov models (HMMs), a class of time-series models that are essentially an extension of mixture models by taking into account temporal dependencies, to analyse such PA time series.

HMMs are now a well-developed area of statistics with books and papers such as [39,40] providing in-depth explanations of the models, from basic definitions to estimation to results. These processes, also known as probabilistic functions of Markov chains and hidden Markov processes, came into prominence in the 1960s by the work of Baum & Petrie [41] and 1970s where [42,43] developed the Baum–Welch algorithm for estimation. Earlier applications of HMMs were in character and speech recognition [44–46], and HMMs have since been used in many different areas such as biology and engineering (see, e.g. [47] for an application in genome sequencing). Regarding accelerometer data, HMMs have been used, among other methods such as Gaussian mixture models and K-means clustering, as an unsupervised machine learning technique for the task of recognition and classification of specific human activity modes such as standing, walking, running, ascending/descending, sitting, cycling etc. This scenario is different to ours, in particular as data acquisition in these cases is much denser at epoch lengths in units of small fractions of a second over a short time span of typically less than an hour, and the research questions is aimed at being able to recognize a specific kind of activity (see, e.g. [48–51] and review in [52]). Similarly, HMMs have been applied to other physiological data acquired at high frequency, in particular the applications to polysomnographic (PSG) recordings, including electroencephalogram (EEG), electrooculogram (EOG) and electromyogram (EMG) signals, by [53–55] to classify and score stages of (rapid eye movement) REM and non-REM sleep, although it is not foreseen that such measurements can be taken for the purpose of long-term monitoring. We shall show that HMMs naturally provide the necessary tools to model the features observed in the type of data we are interested in, and can be further extended toward a dynamic Markov process which is influenced by a circadian oscillator, the strength and nature of which can be inferred from an individual's PA data from a sensing device, that can be unobtrusively worn over many days/weeks.

## 2. Model and inference

**Figure 2.**
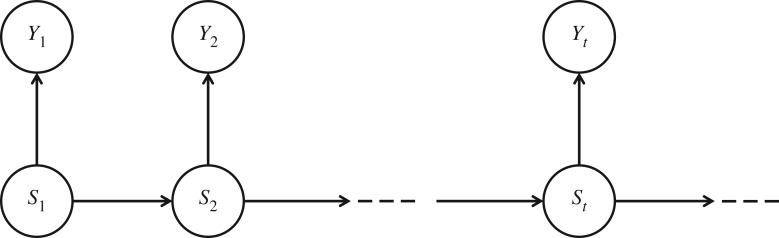
Directed acyclic graph of a hidden Markov model where *S*_*t*_ is the unobservable states and *Y*_*t*_ represents the observable time series.

Let *Y*^(*T*)^ = {*Y*_1_, …, *Y*_*t*−1_, *Y*_*t*_, …, *Y*_*T*_} denote the observations on activity where *t* ∈ {1, …, *T*} and *T* is the sample size. Let *S*_*t*_ ∈ {1, …, *m*} denote the unobserved activity state at time *t*. The notation P( · ) stands for the probability mass function or density function, whichever appropriate. We shall use the short notation *X*^(*t*)^ = {*X*_1_, …, *X*_*t*_} for arbitrary variable *X*. The probabilistic structure of a HMM is represented by the well-known conditional independence graph seen in figure 2, which is a special case of a directed acyclic graph (DAG), and is based on the following two assumptions:

(A1) the sequence of states *S*_*t*_ is a Markov chain satisfying the Markov property: P(*S*_*t*_ | *S*^(*t*−1)^) = P(*S*_*t*_ | *S*_*t*−1_),
(A2) conditionally on *S*_*t*_, the *Y*_*t*_'s are independent and *Y*_*t*_ depends on *S*_*t*_ only: P(*Y*_*t*_ | *S*^(*t*)^, *Y*^(*t*−1)^) = P(*Y*_*t*_ | *S*_*t*_).

It is straightforward to see that the joint distribution of the observations and the hidden states of the DAG is2.1
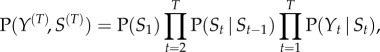
from which the data likelihood can be obtained by summing over the possible combination of states (see [39])2.2
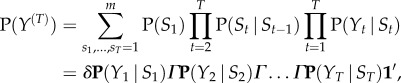
where 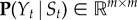 is the conditional probability matrix with *j*'th diagonal entries ***P***(*Y*_*t*_ | *S*_*t*_)_*j*,*j*_ = P(*Y*_*t*_ | *S*_*t*_ = *j*), 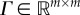 is the Markov chain transition matrix with elements ***Γ***_*j*,*k*_ = P(*S*_*t*_ = *k* | *S*_*t*−1_ = *j*) and whose rows sum to one, 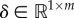 is the initial state distribution and 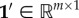 is a vector of ones.

The HMM is hence parametrized by the non-zero entries in ***δ***, ***P***(*Y*_*t*_ | *S*_*t*_) and ***Γ*** which are unknown. Given the output observation sequence *Y*^(*T*)^, the maximum-likelihood estimator of the unknown parameters can be efficiently found, either through direct maximization or based on an expectation maximization (EM) algorithm, called the Baum–Welch algorithm [39], for which closed-form expressions and computationally fast steps exist when the observational distribution ***P***(*Y*_*t*_ | *S*_*t*_) is Gaussian. However, caution is necessary as the likelihood may have local maxima and it is advised to test different starting values for the parameters.

## 3. Application to activity data from healthy subjects

### 3.1. Data and data pre-processing

We shall show results of fitting HMMs to PA count data recorded by the Move3 sensor for 46 healthy individuals (19 male, 27 female; aged 21–75 years, median 35 years) from two countries (UK and France) who volunteered to wear the sensor over a period of 4–7 days. All subjects were asked to keep their usual daily routines, besides carrying the sensor day and night for the whole monitoring duration. In addition to activity, this device also provides 5-min recordings of the skin temperature. Missing values occur as the individuals remove the device to avoid contact with water—typically this happens once a day for around 20 min. The missing values can be marked retrospectively by noting that the contemporaneous temperature records show a sudden decrease towards room temperature. Generally the missing data ratio is around 1%–3% in this dataset. The estimation algorithm is adapted in a straightforward way by propagating the transition matrix corresponding to the last time point preceding the missing values [48].

Collected over many days the data are of considerable size and it is desirable to be able to apply computationally efficient methodologies. In this study, we will assume Gaussianity of the observational densities of the square root transformed 5-min averaged PA count data (see electronic supplementary material, figure S2). We note that the assumption of Gaussianity for the observational densities of the square root transformation corresponds to the assumption of a mixture of non-central chi-square distributions which can account for both, non-negative domain and positive skewness, on the original scale of averaged PA counts.

### 3.2. Number of states

An obvious question is how many states *m* should the model have? The Bayesian information criterion (BIC) suggested models with 2–5 states, with four being chosen in about two thirds of the cases, while Akaike's information criterion (AIC), known to be less parsimonious, tended to generally prefer one more state than the BIC. We speculate that some inter-individual variation in the number of states may be due to the fact that some individuals have a larger range of types of activities than others, however such deductions will largely depend on the measurement process, i.e. the specifications and positions of the accelerometer used, and a controlled study would be needed to substantiate this. Our results on the range of the number of states are in broad agreement with an extensive review [56] which compiled results from more than 40 studies on PA data, covering different types of accelerometers and their position on the body^1^. The majority of these studies developed cut-off points to achieve 2–3 states for adults and older adults. However, one major difference to studies, which derive general cut-off points for PA classification, is that in our study the HMMs produce an individual-specific probabilistic classification of PA. This is in line with our interest in identifying and monitoring the personal long-term circadian rhythm of subjects whose individual lifestyles may be heterogeneous covering a range from sedentary to active. We note that the estimated conditional means and duration of the states can however be used to quantify how active a lifestyle a person leads in comparison to others.

While one needs at least two states to distinguish between rest and activity, the results here consistently indicate that better model fits are achieved if more than two states are assumed. On the other hand, choosing a parsimonious number of states will be more robust to the fact that the day-to-day variability of different particular activities may be substantial and may appear non-circadian despite the presence of a regular circadian rest–activity rhythm. For the purpose of our study, we specified *m* = 3 for all individuals, which not only addresses this compromise in complexity but also achieves a consistent interpretation across all individuals in the sample in that for each individual the lowest activity state corresponds to the prolonged rest period, which normally occurs at nighttime, while the other two stages are predominantly associated with the active daytime.

### 3.3. Parameter estimates

The parameter estimates contain useful interpretable information about the individual's sleep–wake behaviour. We shall discuss typical results of fitting a HMM for two example subjects, 16 and 18, with *m* = 3 states that can be interpreted as inactive (IA) for *S*_*t*_ = 1, moderately active (MA) for *S*_*t*_ = 2 and highly active (HA) for *S*_*t*_ = 3, where *S*_*t*_ = *j*;*j* = 1, 2, 3 also denotes the entry number of the corresponding state in all vectors and matrices.

The estimated model parameters for subject 16 are as follows: the transition probabilities are
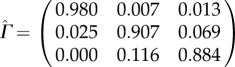
with conditional observation densities for IA state: *Y*_*t*_ | (*S*_*t*_ = 1)∼*N*(0.92, 0.68^2^), for MA state: *Y*_*t*_ | (*S*_*t*_ = 2)∼*N*(3.1, 1.11^2^) and *Y*_*t*_ | (*S*_*t*_ = 3)∼*N*(5.37, 0.74^2^) for HA state. The initial state distribution is 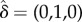, i.e. the initial state is estimated to be MA. For subject 18 the estimated model parameters are:
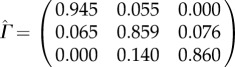
with conditional observation densities for IA state: *Y*_*t*_ | (*S*_*t*_ = 1) ∼ *N*(1.25, 1.26^2^), for MA state *Y*_*t*_ | (*S*_*t*_ = 2) ∼ *N*(6.98, 2.23^2^) and for HA state *Y*_*t*_|(*S*_*t*_ = 3) ∼ *N*(12.06, 0.92^2^). The initial state distribution is 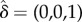, i.e. the initial state is estimated to be HA.

**Figure 3.**
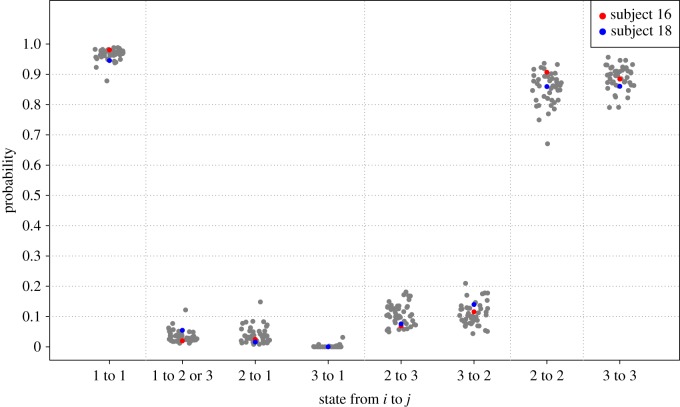
Estimated transition probabilities for 46 healthy individuals. The integers 1, 2 and 3 represent the inactive (IA), medium active (MA) and highly active (HA) states, respectively.

The results for the probabilities in the transition matrix for all 46 individuals are plotted in figure 3. The high probabilities along the diagonal suggest a high chance of staying in the current state which highlights the dependence between successive observations and clearly justifies the use of a time-series modelling approach such as provided by the HMM. The probability is highest for the IA state, as estimated by 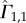, due to the prolonged period of rest at night. The slightly lower values for 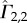 and 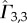, together with the elevated off-diagonal values for 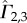 and 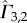, indicate that there is a higher chance of switching between the two active states. In fact, it is these transitions that account for the high variability observed in the data during the day and is due to the fact that people undertake different physical actions in their wake period. The transition from the two active states, HA and MA, to rest is found to happen almost exclusively via the MA state as 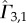 is estimated to be zero, or very close to zero, for all individuals in the sample. A particular transition of interest is from IA to either of the active states with estimated probability 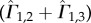, where high values indicate many episodes where the person is likely to have interrupted sleep and one can hence assume that the estimated transition probability from IA to any active state contains information about the quality of sleep of a person. For example, it is estimated to be 0.02 for subject 16 and 0.055 for subject 18, which suggests that subject 18 has experienced about twice as many sleep interruptions as subject 16 during the study time. We found a pronounced positive correlation (0.86 for Spearman rank correlation) between 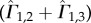 and 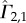, i.e. the transitions into and out of the IA state, due to the fact that subjects who often interrupt their IA state also often attempt to get back into it.

**Figure 4.**
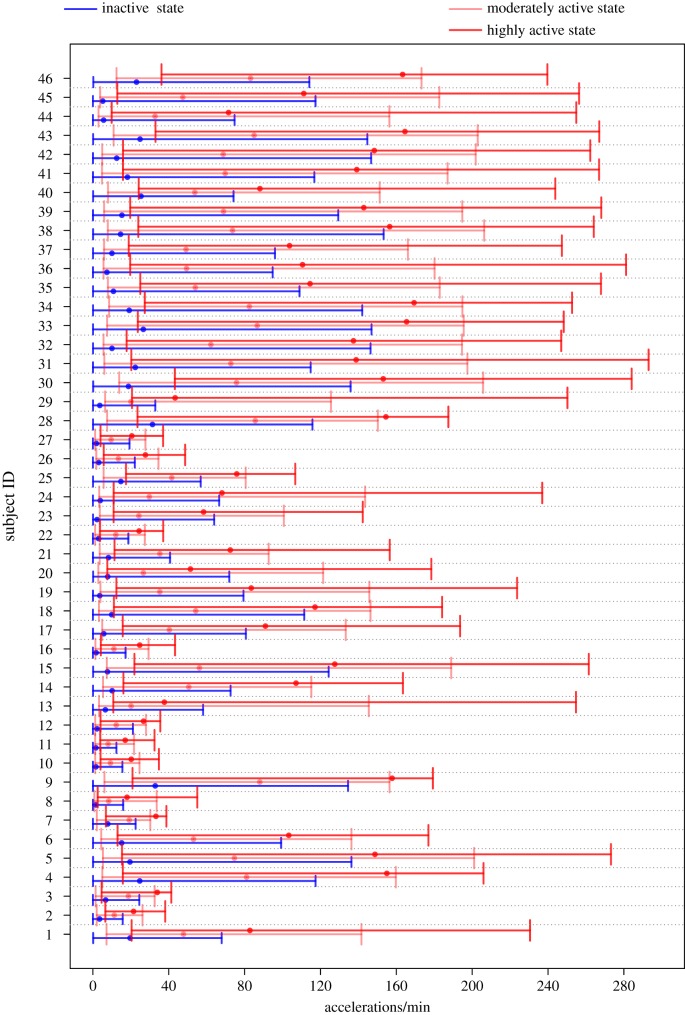
Estimated conditional observation densities. Mean and central 90% range for three states: inactive (blue), medium active (light red) and highly active (red) for 46 subjects. Values on *X*-axis refer to original scale of average PA counts.

The estimated conditional observation densities confirm our visual impression that the IA state has a relatively low value. The MA state usually is characterized by a higher variance also in comparison to the HA state. The latter may be due to the dampening effect of the square root transformation. Figure 4 hence plots the mean and central 90% range of the three estimated observational densities for all 46 individuals, where the results are transformed to the non-central *χ*^2^ distribution on the original scale of the average 5-min PA counts. The mean of the HA state provides an alternative estimator of the *amplitude* without having to rely on Fourier methods. The three states identified by the HMM are specific to the subject and we can see a large variability in the intensity of the three states between the individuals. This may be due to heterogeneous lifestyles regarding activity although it should be noted that such interpretation is likely to also depend on the settings of the accelerometer and where it is positioned on the body^2^.

### 3.4. State estimation

**Figure 5.**
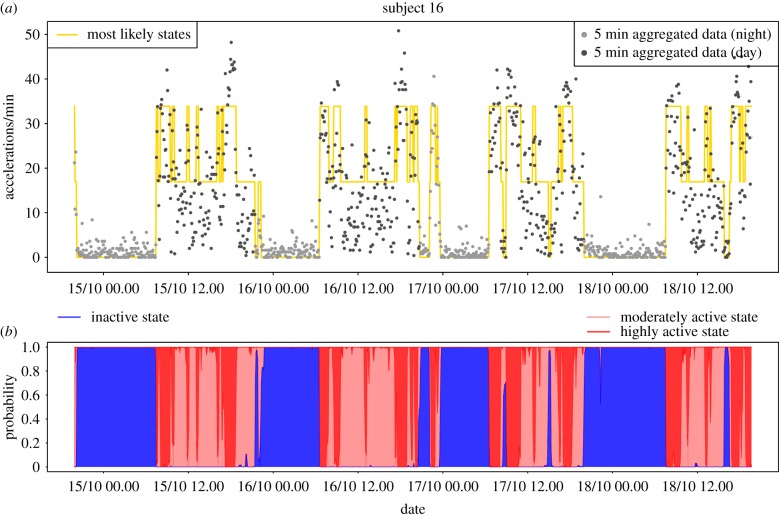
State estimation for example subject 16. (*a*) Time series of activity with yellow line indicating the mostly likely state using local decoding. (*b*) SP plot, i.e. cumulative plot of P(*S*_*t*_ = *j* | *Y*^(*T*)^) for *j* = 1 (IA, blue), 2 (MA, light red), 3 (HA, dark red).

**Figure 6.**
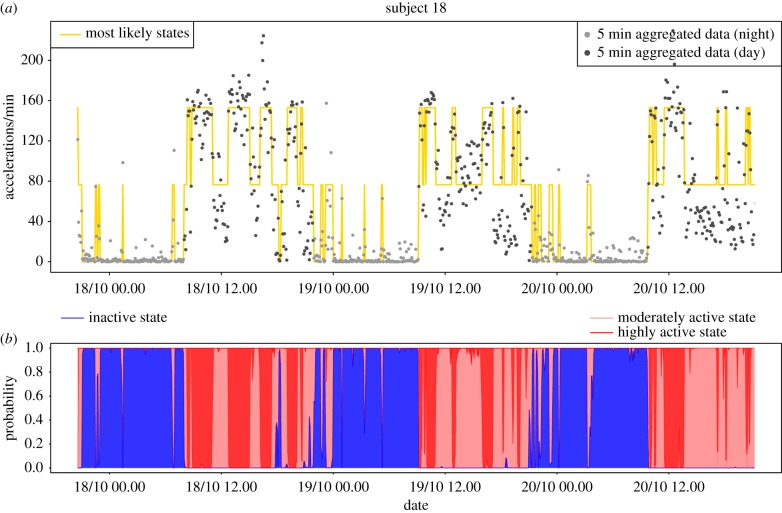
State estimation for example subject 18. (*a*) Time series of activity with yellow line indicating the mostly likely state using local decoding. (*b*) SP plot, i.e. cumulative plot of P(*S*_*t*_ = *j* | *Y*^(*T*)^) for *j* = 1 (IA, blue), 2 (MA, light red), 3 (HA, dark red).

Local decoding [39] can be used to estimate the predicted state at time *t* by
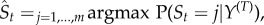
where the estimated conditional state probabilities P(*S*_*t*_ = *j* | *Y*^(*T*)^) are conveniently available as part of the inference algorithm. The predicted sequence of the most likely states for the two example individuals can be seen in figures 5*a* and 6*a*, which show that for both individuals the IA state is predominant at night and that during the day there are many transitions between the MA and HA states. For a more informative visualization, we propose to plot P(*S*_*t*_ = *j* | *Y*^(*T*)^) for *j* = 1, 2, 3 (which add to 1) cumulatively for each *t*, and associate with each state a different colour (blue for IA, light red for MA, dark red for HA). We shall refer to the resulting plot as *state probability* (SP) plot. Figures 5*b* and 6*b* show the SP plots for the example subjects. These diagrams allow us to quickly assess how probable the most likely state is and what other states have noticeable probability and give us visual information on how well a person has rested. In particular, if they have solid blue areas, i.e. rarely move into the active states during night, then we might deduce that the person has obtained a good night's rest, as the example subject 16 seems to have done. In contrast, subject 18 (figure 6) has experienced many interruptions at night which may be indicative of relatively poor quality of sleep.

We may so far conclude that a homogeneous HMM achieves satisfactory model fits (see electronic supplementary material, §1 for details on analysis of pseudo residuals) where estimation results of the parameters are interpretable and the estimated state probabilities from the HMM provide a retrospective analysis of the time-varying characteristics of a person's sleep–wake cycle as they are locally decoded given the observed data, and as such can be used to generate stochastic simulations, e.g. to obtain approximate bootstrap confidence intervals. However, in order to be able to explicitly infer and quantify the circadian nature of the rest–activity cycle we proceed by suggesting a circadian parametrization of the HMM.

## 4. Circadian harmonic Markov model

As in mathematical modelling (see, e.g. [57]) the sleep–wake behaviour is a result of the dynamic behaviour of the model, here it would be natural to assume that the transition probabilities of the dynamic Markov process are influenced by a circadian oscillator where we are interested in estimating the strength and nature of this influence from an individual's activity data. If one views the *m* possible outcomes of the state as a multinomial variable, then the prediction of the probabilities of the different possible outcomes, given a set of independent variables *X*_*t*_, is addressed by the standard multinomial logistic link function. In the framework of HMMs the logistic link between *X*_*t*_ and the entries of ***Γ*** is given by (see [58])4.1
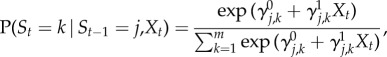
where *γ*^0^_*j*,*k*_, *γ*^1^_*j*,*k*_ are coefficients such that, for identification, one of each per row *k* is fixed, e.g. we set *γ*^0^_*k*,*k*_ = 0, *γ*^1^_*k*,*k*_ = 0, *k* = 1, …, *m*. The entries of the transition matrix each follow a periodic circadian rhythm if *X*_*t*_ is a circadian oscillator, where here we chose the 24-h cosine function as a basis. We did not include any further subharmonics as the number of parameters increases quickly, i.e. proportionally with dim(*X*_*t*_)(*m*^2^ − *m*). We note that the functional form of equation (4.1) is flexible in that even in the case of using only the first harmonic the resulting oscillations in the transition probabilities can be polyphasic and markedly different from simple sinusoidal curves. We shall refer to this modelling approach as the *harmonic HMM* for short. Incorporating a harmonic into the transition probability matrix makes inference a more challenging task as there are no closed-form solutions for the EM algorithm. We proceeded by resorting to numerical optimization methods, where we used the *solnp* routine provided by the R package *depmixS4*^3^.

**Figure 7.**
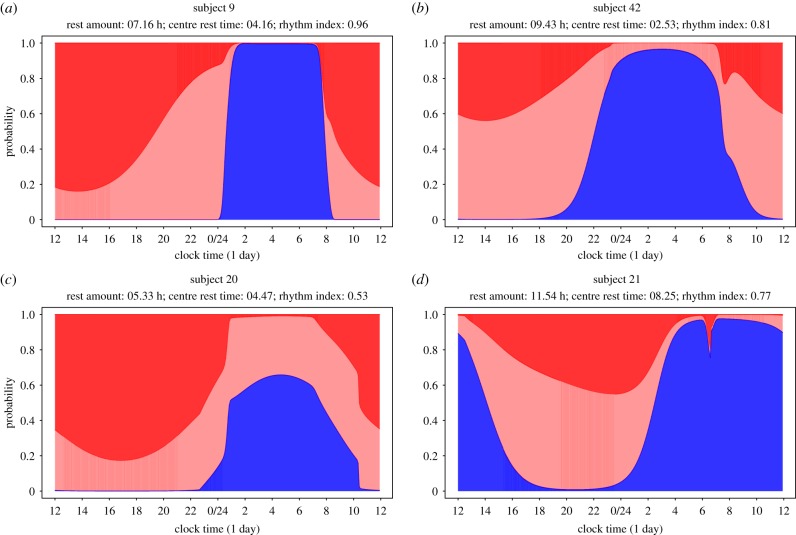
(*a-d*) Circadian state probability plots from harmonic HMM for four example subjects. Each panel shows the periodic time profile of the three state probabilities plotted in cumulative manner analogous to the SP plots above with same colour coding as in previous figure. The estimated values for rest amount, centre of rest and *RI* are stated in the heading of each panel.

While one can in principle study the estimated circadian variation of each probability in the transition matrix, we suggest that the information can be efficiently summarized by plotting the 24-h periodic profile of the state probabilities, which can be computed by applying 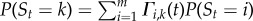 for *i* = 1, …, *m* from the estimated initial state, in a manner analogous to the SP plots above. Figure 7 gives four examples of the resulting plots which give a precise summary of a subject's typical daily profiles of the circadian state probabilities:

The prolonged period of rest starts during 00.00–01.00 and finishes around 08.00 with no noticeable probability of interruption for subject 9 (*a*). The sharp incline as well as decline of the blue profile at the start and end of the rest period are strongly indicative of the subject's starting and finishing their rest at a regular time every day. Subject 42 (*b*) has a less regular start of the resting time as the probability of rest increases gradually after dinner time. This is in line with the person having reported that she watched TV for several hours every night. The subject has a probability of around 5% of interrupting their rest during the night. The subject is also likely to have used an alarm clock, set at around 07.00, when there is a sudden increase of the HA probability. However, one can also also discern a weekend effect where the subject did not use the alarm clock and tended to sleep longer. Subject 20 (*c*) has a substantial probability of 40–50% of interrupting their night rest and obtains only an amount of about 5.5 h of rest as can be estimated by the size of the blue area. This subject reported to have taken antidepressant and anti-anxiety medication, and the profiles indicate that this person is suffering from a highly interrupted rest pattern at night and a lower total amount of rest. Finally, subject 21 (*d*) can be seen to have experienced an extraordinary large amount of rest (over 11 h) which is centred around 08.00 as the individual starts resting very late and rises in the afternoon. This subject reported to have taken an anti-depressant and a sleeping pill which is particularly known in the press to leave people drowsy in the morning. Generally, we can deduce from the more gradual changes in the IA probability at the start and end of the rest periods that subjects 42, 20 and 21 all start and finish their rest period at more irregular times and have a more interrupted resting pattern than subject 9. The circadian probability profiles for all 46 individuals in the sample can be inspected in the electronic supplementary material, figure S5.

## 5. Circadian parameters

**Figure 8.**
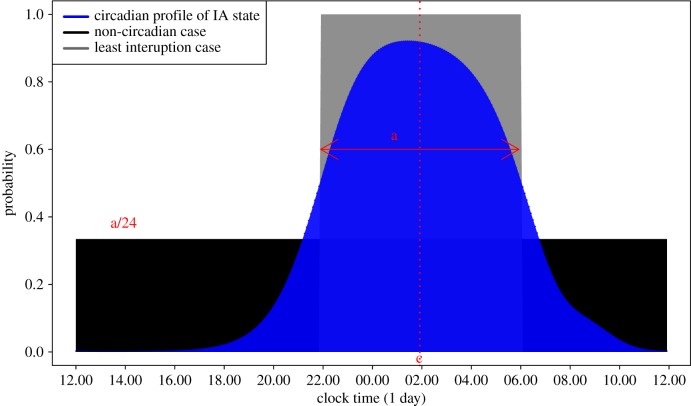
Diagram for deriving rhythm index *RI*. Blue area gives the probability of IA state as in previous plot for a subject with an amount of rest *a* = 8 h (equal to the size of the blue area) centred at *c* = 02.00. The grey rectangular area corresponds to the probability of IA state under a perfectly regular circadian rhythm of rest for which the *RI* assumes a value of 1. The black rectangular area corresponds to probability of IA state in the absence of any circadian rhythm with an *RI* value of 0. Note that the size of the grey and black areas (they are both over-plotted by blue) correspond to a total rest time of 8 h.

The harmonic HMM provides a model on the basis of which statistics can be derived that quantify an individual's rest–activity rhythm. The *amount of rest*, *a* (hours) say, can be estimated by the blue area or integral under the function *P*(*S*_*t*_ = 1), while the midpoint or *centre of rest*, *c* say, corresponds to the gravity centre of the blue area. Furthermore, given a subject's values for *a* and *c*, one can construct an index on the unit interval, which assumes a value of 1 for the strongest circadian rhythm characterised by a rest period with no interruptions, i.e. *P*(*S*_*t*_ = 1) = 1, and perfectly regular start and end times at [*c* − *a*/2] and [*c* + *a*/2], respectively, as shown by the rectangular profile of width *a*, centred at *c* in figure 8. On the other hand, a complete lack of circadian rhythm refers to the case where the probability of rest is constant and hence equal to *a*/24 (see black shaded profile in figure 8). Typically an individual's profile will lie between these two extreme cases, and we can therefore construct a rest–activity *rhythm index* *RI* such that 0 ≤ RI ≤ 1 where5.1
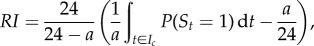
where *I*_*c*_ = [*c* − *a*/2, *c* + *a*/2]. It is easy to verify that *RI* assumes a value of 1 when 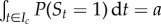, i.e. the strongest case of circadian rhythm, and a value of 0 when 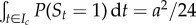, i.e. absence of circadian rhythm. In our 46 healthy subjects, *RI* ranges from 0.47 to 0.96 with a median of 0.78 (see electronic supplementary material, figure S6 for a plot of *RI* values against each subject ID). Returning to the four examples in figure 7 we note that subject 9 has an *RI* of 0.96, the highest value in our sample, while subject 20, has an *RI* of 0.53, the second lowest value in our sample.

**Figure 9.**
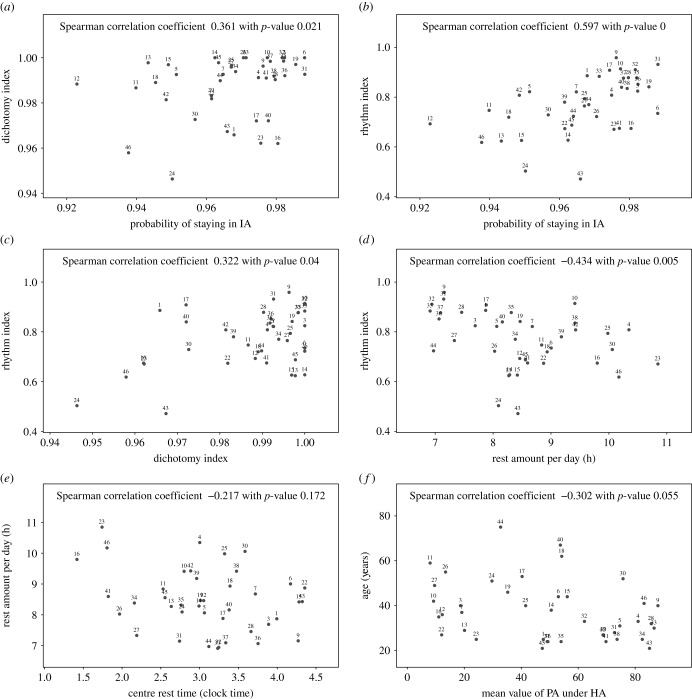
Scatterplots and correlations between estimated parameters: for all individuals (excluding outliers): (*a*) transition probabilities of staying in IA and dichotomy index I < O; (*b*) transition probabilities of staying in IA and *RI*; (*c*) *RI* and I < O; (*d*) rest amount per day and *RI*; (*e*) centre of rest and rest amount per day; (*f*) mean value of PA conditioned on HA state and age. Results of Spearman's rank correlation are reported on each graph. Statistically significant correlations at *p*-value ≤ 0.1 are found for all relationships shown except (*e*).

We computed the various circadian parameters discussed above, as well as the conditional mean value of the HA state, which serves as an estimate of the amplitude, and the *dichotomy index* I < O for all 46 subjects and investigated simple correlations (see figure 9) where five subjects were treated as outliers due to their irregular rhythm and were omitted from the correlation analysis^4^. Significant and interpretable correlations are found between them. As can be expected there is clear positive correlation between the probability of staying in IA and the *RI* (figure 9*b*) as both measures decrease when the subject suffers from sleep interruptions. They are both positively correlated with the *dichotomy index* I < O (figure 9*a*,*c*). The dichotomy index I < O is widely used, but requires the PA data to be partitioned into prolonged IA and active periods. Such a classification depends on the clustering algorithm used, where we provide a clustering algorithm for this in the electronic supplementary material, §2. However, it is not rare that individuals have one or more episodes of rest interrupted by activity at times that are close to the main rest period (for instance, by sleeping in front of TV in the evening). The question of whether or not these episodes are part of the night rest is not clear-cut, so that different values of the dichotomy index I < O may be obtained depending on the outcomes of hand-tuning the clustering algorithm. The *RI* proposed here is similar in spirit to the I < O but can be estimated in a more straightforward way on the basis of the harmonic HMM and we are planning to compare both indices in future work.

**Figure 10.**
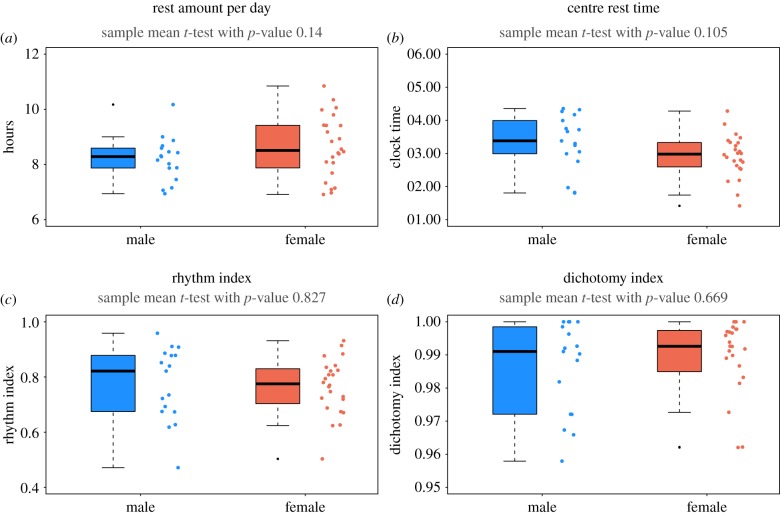
Checking for sex specific effects: boxplots of amount of rest per day (*a*), centre of rest (*b*), *RI* (*c*) and dichotomy index I < O (*d*) for all individuals (excluding outliers) grouped into male (blue) and female (red). The single estimated values for each individual are plotted alongside the boxplot to better show the distribution. Results of *t*-tests for equality of mean between the two groups are reported on top of the graph. There is no significant evidence in this sample in favour of a sex-specific effect in these parameters although we note that the lower *p*-values in the panels (*a*,*b*) suggest that a significant sex-specific effect might emerge for a larger sample size.

An interesting negative correlation was found between the amount of rest and the *RI* index (figure 9*d*) which indicates that a person with a good rest–activity rhythm seems to require less rest. Figure 9*e* indicates that the rest amount is negatively correlated with the centre of the rest time. However, the latter correlation is not significant and a larger sample size would be needed to substantiate this trend. We also found that the mean of HA state is negatively correlated with age, as shown in figure 9*f* which is in line with elderly people being less active. An emerging correlation was detected between the *RI* and the mean of the HA state, or amplitude of activity, with (*p*-value 0.09, plot not shown here) and between the *RI* and amount of HA, as estimated by the size of the dark red area scaled by the expected HA counts (*p*-value 0.06, plot not shown) indicating that more physical exercise might lead to a better rest, but again a larger sample size would be needed to substantiate this. We also considered differences in sex (figure 10) between our rest–activity rhythm related parameters, using simple two-sample *t*-tests, but did not find significant differences although it should be noted that some lower *p*-values were found indicating that females tend to have a slightly larger amount of rest with a slightly earlier midpoint. A larger sample size would be needed to investigate this further.

## 6. Cancer patients on chronotherapy

We are interested in the circadian rhythm of cancer patients and how it is affected by the administration of anticancer drugs which are characterized by a high degree of toxicity [59]. In a clinical context, the European project inCASA [14] obtained, amongst other variables, recordings of rest–activity of cancer patients receiving a multidrug chemotherapy at home [60,61] where the activity of patients with any cancer requiring chemotherapy was recorded every minute with a wrist-watch accelerometer (ActiGraph, Micro MotionLogger, Ambulatory Monitoring Inc, Ardsley, NY, USA), and data were transmitted daily to a server via a dedicated platform installed in the home of the patient. The patients were subject to a chronotherapy, i.e. a chronomodulated delivery of chemotherapeutic agents, while in their own environment.

**Figure 11.**
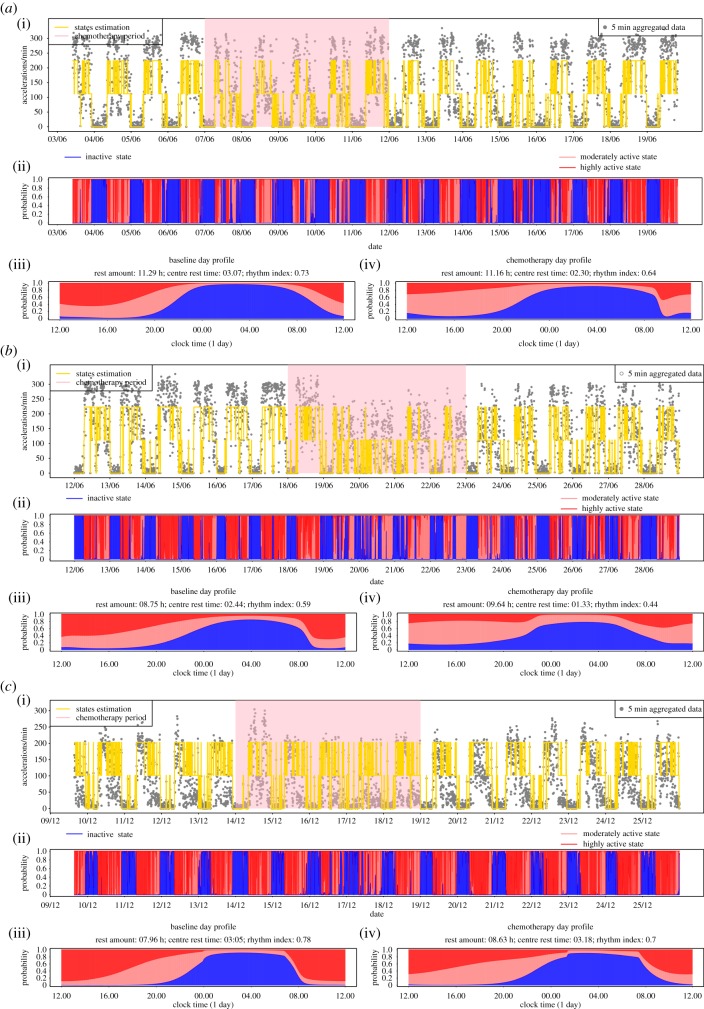
Data and Results for fitting circadian harmonic HMM for three cancer patients (*a-b*). Coefficients for the circadian transition probabilities are assumed to be shifted during chemotherapy as in equation (5). For each patient: (*a*(i)–*c*(i)) time series of activity with yellow line indicating the mostly likely state using local decoding. The highlighted pink section indicates when the chemotherapy was administered. (*a*(ii)–*c*(ii)) SP plot. (*c*) Circadian profile of state probabilities during non-chemo time (*a*(iii)–*c*(iii)) and chemo time (*a*(iv)–*c*(iv)). The estimated values for rest amount, centre of rest and *RI* are stated in the heading of each panel.

We fitted a circadian harmonic HMM with *m* = 3 to the square root of the 5-min averaged PA counts, which was extended by adding, subject to an indicator function *I*_*t* ∈ *T*_chemo__ that equals one during the time of the chemotherapy treatment *T*_chemo_, a second circadian harmonic whose Fourier coefficients, and hence amplitude and phase, may assume different values during *T*_chemo_, i.e. we set6.1
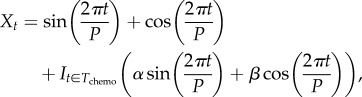
where *P* = 24 h and *α* and *β* are arbitrary coefficients that capture changes in the associated *γ* coefficients in equation (4.1) for the time of the chemotherapy. Figure 11 gives results for three patients, A, B and C, on chronotherapy each with over 2 weeks of PA recordings from a wristwatch. We can see that, before the chemotherapy, the circadian rhythm for patients A and C is relatively regular and their baseline values for *RI* are well compatible with the healthy individuals above. There is evidence of a more sedentary behaviour during the treatment in that patients tend to replace higher by lower activity levels along with elevated probabilities of daytime rest. All three patients have a decline in their *RI* although this is least pronounced for patient C who has the highest baseline *RI* value among the three patients and whose *RI* decreases from 0.78 to 0.70 which is due to some deterioration occurring during the later phase of the chemo. Patient B, whose baseline *RI* of 0.59 is already low in comparison to our healthy individuals, experiences the most pronounced decrease towards an *RI* of 0.44 which is predominantly due to a more interrupted night rest. The SP plots for A and B indicate that the circadian interruption might last until several days after the chemotherapy and future work of ours will consider how to assess whether a patient returns to their baseline circadian rhythm and the length of time that this may take.

These examples show that the harmonic HMM can be extended to quantify changes in the spectral behaviour of the circadian rhythm in PA due to external covariates such as treatment time. Although we have not demonstrated this here, an analogous approach as above may be taken to draw inference about a weekend effect. One can hence further envisage that, on the basis of such a routinely estimated circadian HMM, it will be possible to construct a system that can monitor, in real time, the patient's circadian rhythm during and in-between chemotherapeutic courses taken in the patient's home or own environment. Such a monitoring system could be instructed to issue warning signals to a health expert if the patient drastically changes rhythm or does not return to the baseline rhythm after a treatment course.

## 7. Summary and discussion

In this paper we propose the use of a HMM approach which can address the challenges of modelling activity data, and provides a natural framework for extracting information from them. The model can capture the characteristic features discernible in time series of activity measured over days, such as the notable square wave form with heterogeneous ultradian variances over the circadian cycle of human activity. The estimated parameters can be used to characterize the individual of rest–activity pattern and to study the inter-individual variability. We have further proposed a circadian harmonic HMM which incorporates a circadian oscillator, the nature and strength of which can be quantified from an individual's observed PA data, and plots and parameters, in particular a novel *RI*, that are of interest and relevant to circadian monitoring of human activity over many days. The possibility of assuming that the state transition probabilities may change over time according to covariate information and that the response may be multidimensional allows for a wide range of further modelling approaches with a potential to deal with the multivariate complex and large physiological data sets that may in the near future be acquired regularly and cheaply due to the rapidly developing technology of wearable devices [20]. Parameter inference via maximum likelihood requires the use of optimization procedures for which computationally accessible methodologies exist at least for some standard distributional choices. We note that we have assumed Gaussianity for a suitable transformation of the data and hence our HMMs were relatively easy to implement in particular since some R packages such as (*HiddenMarkov* and *depmixS4*) are already available.

Activity counts taken at very short lengths of epochs display a large proportion of zero and low integer counts during the prolonged IA states. Hence the development of estimation algorithms for mixtures of zero-inflated discrete distributions and Gaussian distributions for the active states may provide an interesting avenue to pursue in order to deal with shorter epoch lengths. However, as Bai *et al.* [21] point out, there are also significant differences in the computation of PA counts between manufacturers and even for new devices from the same manufacturer. Wearable devices are developing rapidly gaining increasing market attention via smart watches, mobile phones and bracelets where there is currently no consensus about their quality in assessing activity duration and sleep quality [62]. Activity recordings mark the beginning of sleep periods by immobility of the subject and therefore tend to overestimate sleep and underestimate wake time [2,63] in comparison to polysomnography (PSG), the current gold standard for measuring sleep, which will mark the onset of sleep through changes of electrical activity patterns in the brain. Hence, the accuracy of activity recordings obtained by accelerometers in measuring actual sleep continues to be investigated [64,65]. Migueles *et al.* [56] uncover significant effects on data comparability with respect to placement, epoch length, sampling frequency, frequency setting of the filtering process that selects the acceleration measured and treatment of missing data (usually due to removal to avoid contact with water) for different generations of accelerometer devices. Although it cannot address differences in the quality of data resulting from different types of measuring devices, an advantage of the HMM approach lies in its ability to translate the information from the observed data into probabilities of being in, and transferring between, different activity states allowing, in principle, for a comparison between studies that may be based on fundamentally different ways of measuring activity. Furthermore, the HMM approach solves the problem of ‘thresholding’ activity into different states in an appropriate way through a probabilistic model whilst respecting the dependencies in time which is a fundamental property of the observed time-series data.

Although we have not discussed this in detail here, it is important to note that the HMM provides a model on the basis of which one can compute theoretically justified confidence intervals which quantify the individual-specific variability of estimated model parameters. Moreover, the estimated harmonic HMM provides a simulation model for realistic data that can, for instance, be used to compute approximate confidence intervals for any non-parametric statistics of interest, such as the I < O or the *RI* developed here. This is an important task essential to evaluating the risk associated with the use of any such statistics for therapeutic treatment decisions in clinic. Such appropriate statistical modelling approaches are needed and the whole approach has the potential to serve as routine model in an online monitoring system that could be implemented to monitor, in real time, the daily rhythm of a cancer patient during chronotherapy in their own home.

Further data are foreseen to be collected as part of an e-health circadian platform created by the French project PiCADo which allows automatic and non-invasive monitoring of circadian biomarkers in cancer patients receiving treatment in their own environment. The platform and the HMM modelling approach suggested here are currently being used and further developed by us in two projects namely to investigate circadian rhythm in cancer patients and in night-shift working individuals.

## Supplementary Material

Supplementary Material

## Endnotes

1 The majority of accelerometers in these studies were positioned on the hip, four studies used the non-dominant wrist.

2 For example, a person who often moves their wrist rather than the central body will appear more active if the accelerometer is positioned on the wrist.

3 Convergence should be assessed for different starting values.

4 The five outliers are subjects 2 (travelled between Europe and US), 8 (suffered from insomnia), 20 (suffered from depression and anxiety), 21 (suffered from insomnia and depression) and 29 (student working at night).

## Data accessibility

R-codes to obtain results for homogeneous and harmonic HMMs along with three example data sets are available at https://github.com/huang1010/hmms.
