# Supplementary material for: Hidden Markov models for monitoring circadian rhythmicity in telemetric activity data
Source: J R Soc Interface. 2018 Feb 7;15(139):20170885. doi: 10.1098/rsif.2017.0885 (PMC5832732; doi:10.1098/rsif.2017.0885)
Supplement: Supplementary Material [file rsif20170885supp1.pdf]

Supplementary Information

Q Huang, D Cohen, S Komarzynski, X-M Li, P Innominato, F Lévi and B Finkenstädt

Hidden Markov Models for monitoring Circadian Rhythmicity in Telemetric Activity Data

Journal of the Royal Society Interface

---

1. Model fitting and pseudo-residuals

As described in Zucchini and MacDonald (2009) the HMM fit can be checked by analysing the pseudo-residuals. Consider the cumulative distribution function (CDF), F, of a random variable Z. Then we have that $F(Z)$ is uniformly distributed on the unit interval. Let $\Phi(\cdot)$ be the CDF of standard normal distribution. Then $\Phi^{-1}(F(Z))$ follows a standard normal distribution, i.e. $\Phi^{-1}(F(Z)) \sim N(0,1)$. Now define the normal pseudo-residuals as

$$u_t = \Phi^{-1}\left(F_{Z_t}(z_t)\right)$$

where z_t denotes the observations. If the model fits well, u_t will be distributed standard normal. In the context of HMMs, the ordinary pseudo-residuals are used to check the general model fit using

$$u_t = \Phi^{-1}\left(\mathrm{P}\left(Z_t \leq z_t | \mathbf{Z}^{(-t)}\right)\right).$$

We perform model checking by looking at the Quantile-Quantile (Q-Q) plot of u_t against a standard Normal distribution. Note that computing pseudo-residuals for the harmonic HMM is considerably more involved due to the time-varying transition matrix and is not provided by any R-package.

As discussed in the main paper, we assume Gaussianity of the observational densities of the square root transformed 5-min mean aggregated PA count data. Supplementary Figure 2 below shows histograms and Q-Q plots of HMM fits when only 5-min averages are computed (left), and when, furthermore, the square root transformation is applied (right). Gaussianity leads to computational efficient inference algorithm, which is of importance when we need to deal with large amount of data for long-term monitoring of circadian rhythm of activity. Q-Q plots for all 46 healthy subjects can be found in the supplementary Figures 3 and 4 below, where in all cases 5-min average and square root transformation are applied.

2. Clustering algorithm to identify prolonged active and inactive periods

Consider we have observations $Y_t, (t = 1, 2, \ldots, T)$, with sampling frequency s_f. The most likely states sequence has been obtained using Baum-Welch algorithm. Define a variable $\widetilde{S}_t$, where $\widetilde{S}_t = 0$ if the inactive state is most likely and $\widetilde{S}_t = 1$ if either of the active states are most likely. The following clustering algorithm is easily coded and can be used to pick prolonged active and inactive periods:

Model Clustering

Input parameters (which can be adjusted according to individuals):
$T_1 = s_f P_1$, $T_2 = s_f P_2$ and $T_3 = s_f P_3$, where
P_1 : each cluster covers at least P_1 hours,
P_2 : clustering in each time point considers the previous P_2 hours mostly likely states,
P_3 : clustering in each time point considers the next P_3 hours mostly likely states.
Steps:
1) For $t < T_1$, assign Y_t to cluster 1, $C_t = C^n$ where $n = 1$.
2) For $T_1 < t < T - T_3$:
   a) If the number of points in C_{t-1} is less than T_1,assign Y_t to cluster C_{t-1}, else:
   b) Compare $\widetilde{S}_t$ with its previous T_2 values. If $\widetilde{S}_t = \text{mode}(\widetilde{S}_{t\in[t-T_2:t-1]})$, assign Y_t to cluster C_{t-1}, else:
   c) Compare $\widetilde{S}_t$ with its later T_3 values. If $\sum_{i=t+1}^{t+T_3} \delta\left(\widetilde{S}_i - \widetilde{S}_t\right)/T_3 \leq 0.5$ where $\delta(\cdot)$ is the indicator function, assign Y_t to cluster C_{t-1}, otherwise assign Y_t to a new cluster $C_t = C^{n+1}$.
3) For $t > T - T_3$, assign Y_t to cluster C_{t-1}
Now we have N clusters $\left[C^1, C^2, \ldots C^N\right]$.
4) If the mode of $\widetilde{S}_t$ in a cluster C^n $(n = 1, 2, \ldots, N)$ is equal to the mode $\widetilde{S}_t$ in the previous cluster C^{n-1}, combine the clusters into one cluster.
5) Suppose have N' clusters $\left[C^1, C^2, \ldots C^{N'}\right]$.
   For the clusters with the mode of $\widetilde{S}_t$ being the inactive state, choose the first and last time point with this cluster as the start and end of an inactive/rest period.
Output: estimation of prolonged inactive period time points.

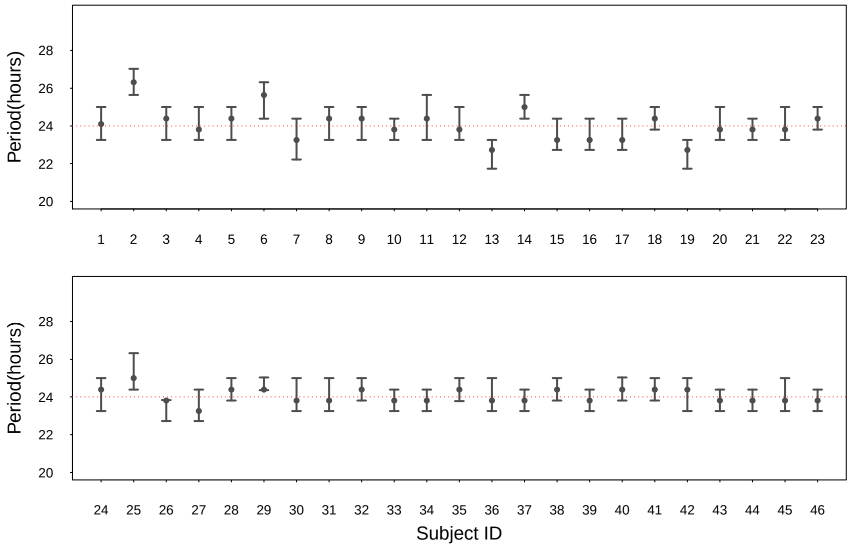

Figure 1. Estimated dominant period length, along with bootstrap 90% confidence intervals (see Costa et al. (2013) for methodology), for all 46 individuals analysed in main paper.

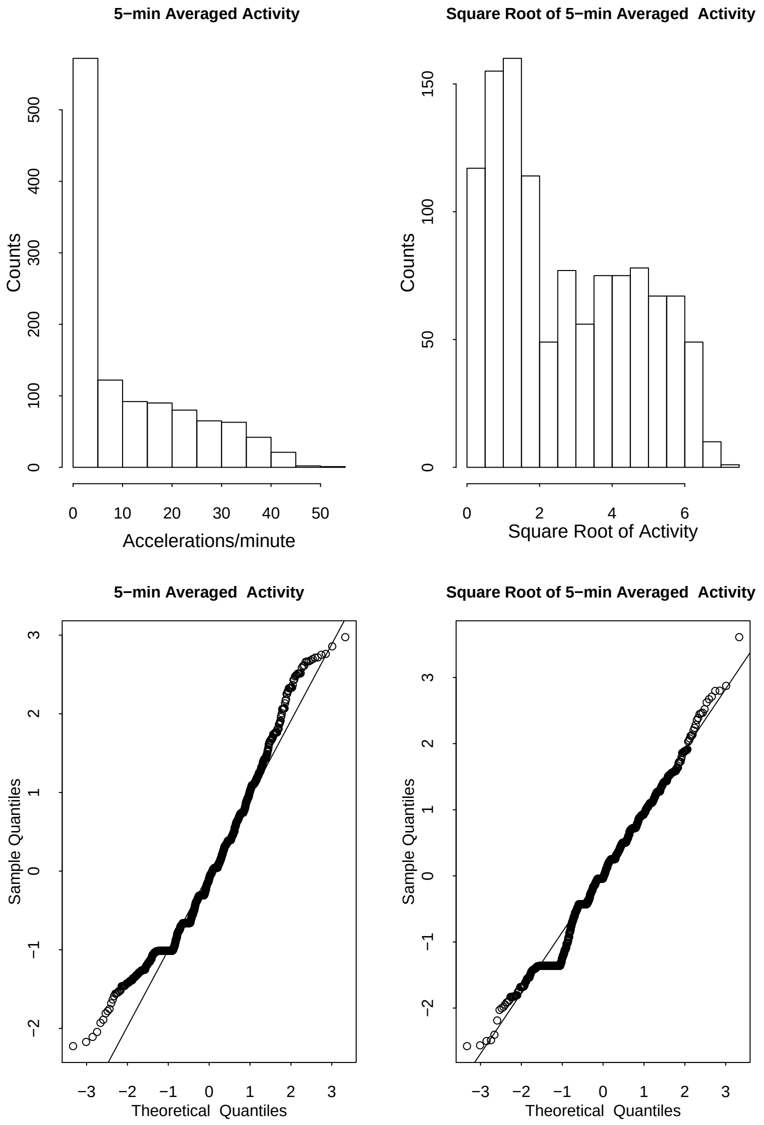

FIGURE 2. Histograms and QQ-plots of an example subject 16. (a) Top left: Histogram of 5-min averaged time series data; (b) Top right: Histogram of 5-min square root transformed time series data; (c) Bottom left: Q-Q plot of pseudo residuals from HMM fit with $m = 3$ using 5-min averaged activity (Jarque-Bera test has $p-$value < 0.001 i.e. normality is rejected); (d) Bottom right: Q-Q plot of pseudo residuals from HMM fit with $m = 3$ using square-root of 5-min averaged activity, with Jarque-Bera test for normality $p-$values 0.19 i.e. normality is accepted

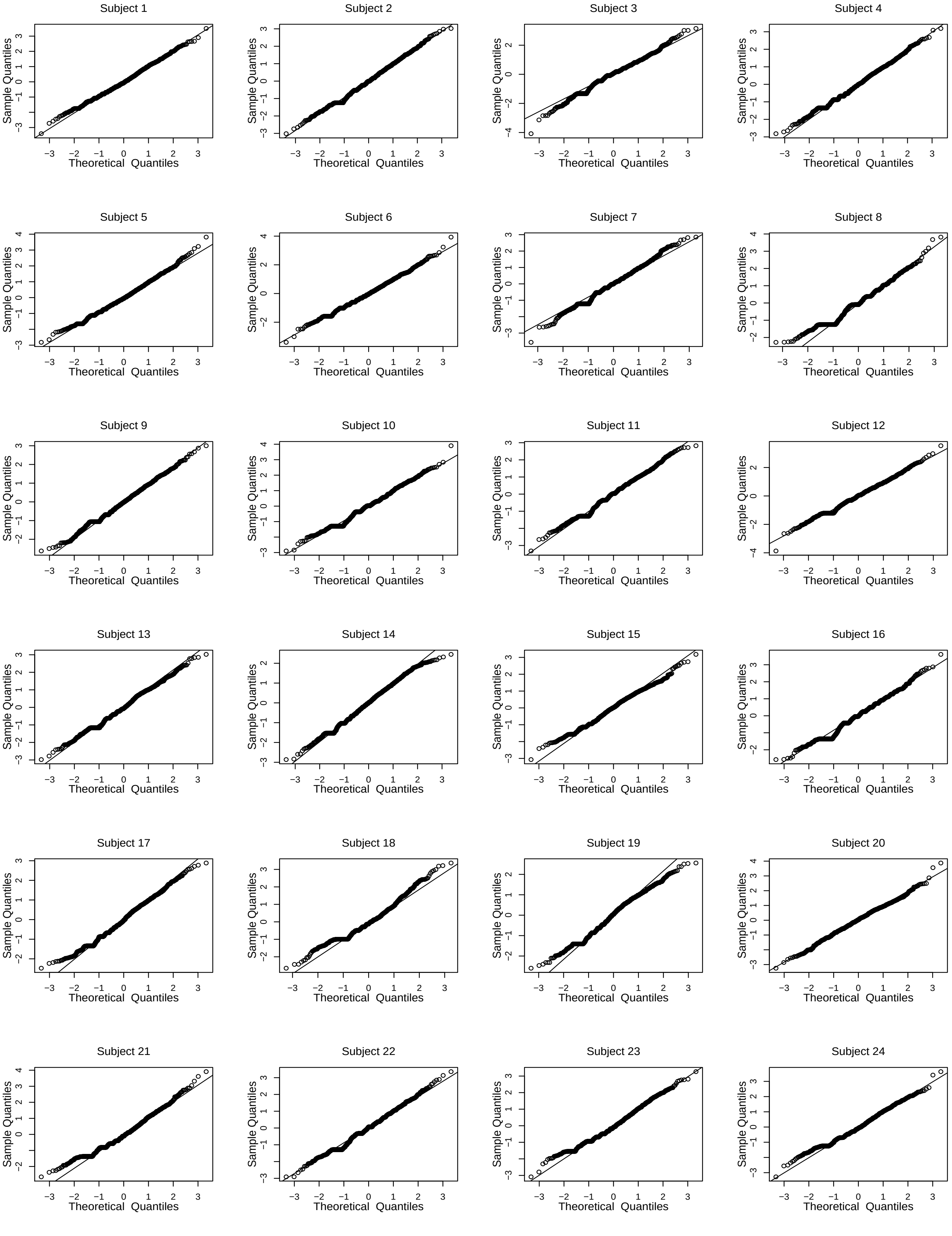

FIGURE 3. (Q-Q) plots of the pseudo-residuals for healthy subjects 1-24, three states are assumed and square-root of 5-min averaged activity is applied.

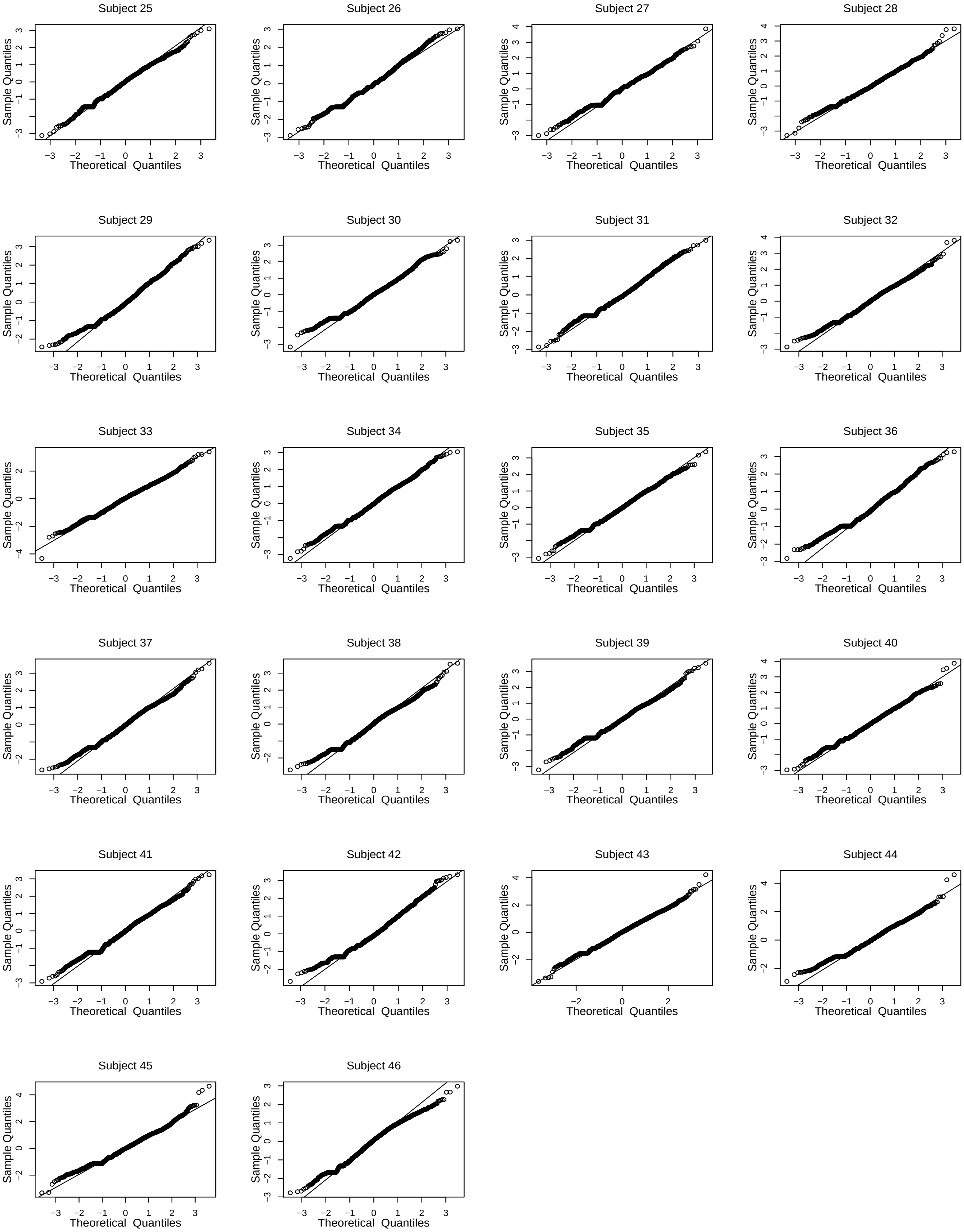

FIGURE 4. (Q-Q) plots of the pseudo-residuals for healthy subjects 25-46, three states are assumed and square-root of 5-min averaged activity is applied.

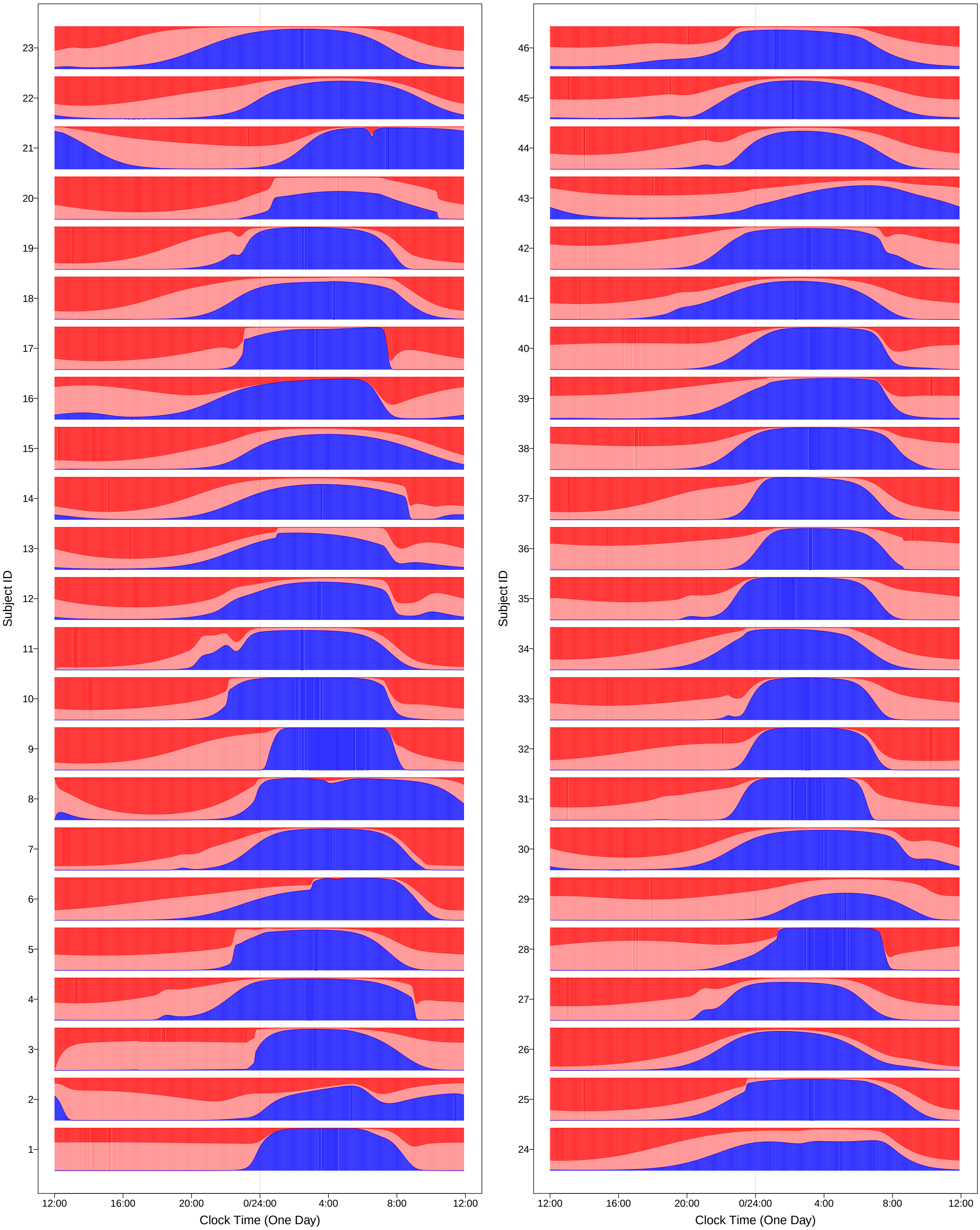

FIGURE 5. State probability (SP) plots resulting from 24h-harmonic HMM for all 46 healthy individuals in sample. One can distinguish between the various types of circadian rhythmicity such as early risers (for example individual 9, 17 and 31), late types (individual 8, 21 and 43) or individuals who experience a lot of interruptions at night (for example subjects 20, 24 and 29).

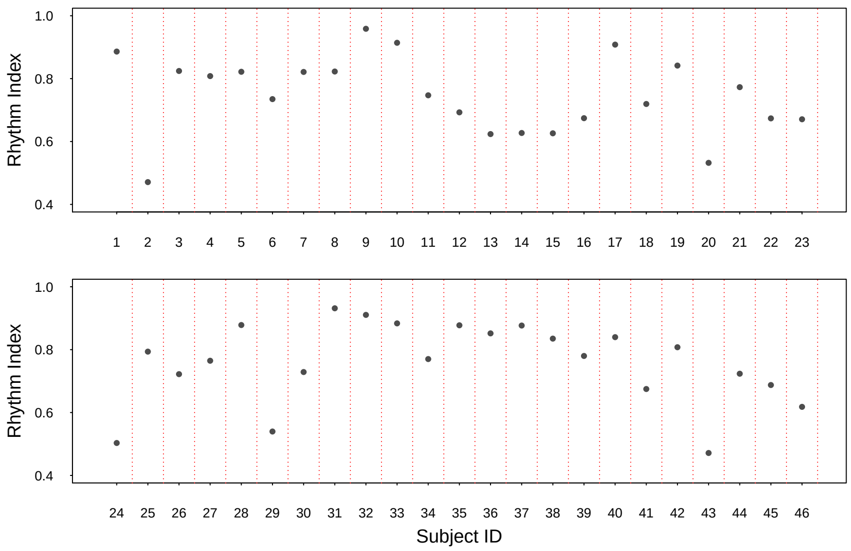

FIGURE 6. The estimated rhythm index RI for all 46 healthy individuals in sample.
